# A Conversation with Renee Wegrzyn

**DOI:** 10.1021/acscentsci.3c00245

**Published:** 2023-03-03

**Authors:** Dalmeet Singh Chawla

In September 2022, U.S. President Joe Biden appointed applied biologist Renee Wegrzyn as the inaugural director of the Advanced Research Projects Agency for Health (ARPA-H). Biden and Congress have shown a lot of faith in the effort, making $1.5 billion available to the agency in 2023.

**Figure:**
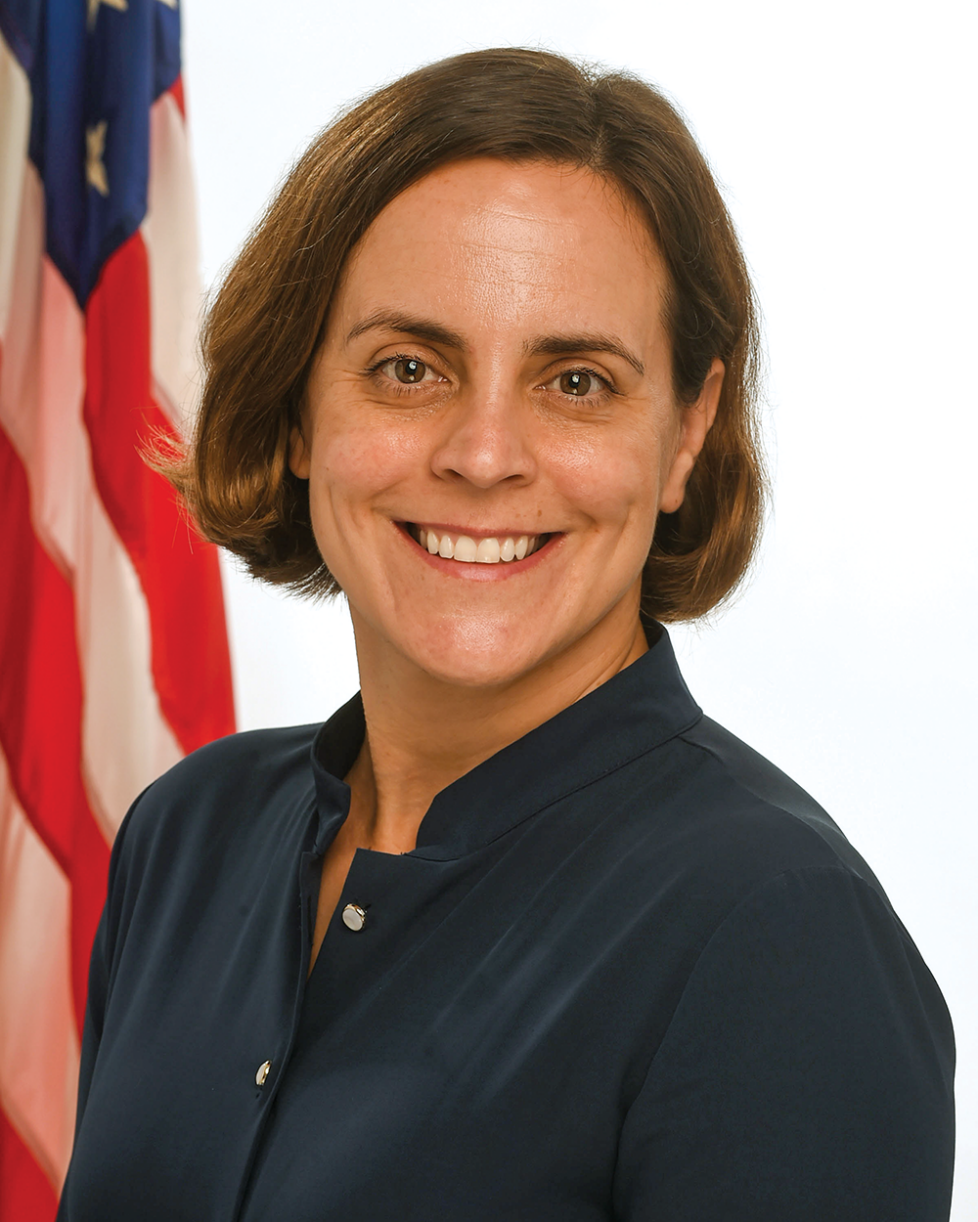
Credit: DARPA

The new agency—inspired by the Defense Advanced Research Projects Agency (DARPA), Intelligence Advanced Research Projects Activity (IARPA), and Advanced Research Projects Agency-Energy (ARPA-E)—was launched in March 2022 with the goal of pushing the boundaries of innovation in health and biomedical research. Like previous ARPA efforts, ARPA-H aims to fund high-risk, high-reward projects that may not be likely to attract funding under traditional schemes.

Wegrzyn comes to ARPA-H with experience in government and out. In 2020, after a 4-year stint at DARPA, she became the vice president for business development at Ginkgo Bioworks, a biotechnology firm specializing in producing genetically engineered bacteria with industrial applications.

Dalmeet Singh Chawla spoke with Wegrzyn about projects ARPA-H plans to fund and about her vision for the future of health care. This interview was edited for length and clarity.

## What was ARPA-H made to do, and what are going to be your first steps building the agency?

The agency was created because there was a place in the ecosystem for high-risk, high-payoff research and development programs. There were some examples of small-scale programs that already did this, but ARPA-H programs will focus on big-scale projects, ranging from $50 million to $150 million, to have a crack at solving some of the biggest issues in health.

In health care, there’s often urgency to bring solutions forward quickly. Our agency is designed to do exactly that. My priority right now is to identify around 20 program managers who are in academia, industry, or other parts of the government and have big ideas to solve big problems and a solid track record. They’re likely known in their community and have interdisciplinary skills, because the big problems cannot be solved in silos.

The program managers really have to have a hands-on approach—we call it a high-contact sport—including traveling to visit their grantees. What success looks like is that we create this environment where we allow them to be dreamers and go big on these projects and give them some room to fail.

## Similar agencies, including DARPA, have been around for a while. Why has your agency been founded now?

I can speculate. Around a decade ago, the Biological Technologies Office launched within DARPA itself. So there’s been a growing understanding across the biotech sector, and even in other sectors, including defense, about what a big role biology and health are going to play moving forward. In the past, I also worked as a program manager at DARPA, overseeing projects on using organisms creating new molecules, gene-editing technologies, and biosurveillance.

With the pace of innovation but also new challenges that we face in health, whether it is pandemics or the climate crisis, now is really the time to move forward and launch this organization with a very specific focus on health.

## What areas will ARPA-H target?

We’re not prescribing what programs will be carried out, but we have decided on four key areas that we'll focus on: “health science futures,” where we expand what’s technically possible across different areas by building new tools and platforms; “scalable solutions,” in which we make sure innovations reach the public quickly and efficiently; “proactive health,” which are preventative programs in the viral, bacterial, chemical, physical, or psychological sciences that reduce the likelihood of Americans becoming patients; and “resilient systems,” where we build integrated health systems to endure crises like pandemics, social disruption, climate change, and economic instability.

Also, tools and platform technologies are really a brilliant path forward because we can reuse a lot of those capabilities when we make the next medicine and the next vaccine. Greater investment is really needed, especially when we think about things like rare diseases where so much goes into developing a new drug and it treats only a small number of people. But if we can find commonalities, we can create platforms that can then be used for a number of things. This changes the incentive structure of how [pharmaceutical companies] may pursue some of those drugs, so I’m very bullish on that side of the house, on big biotechnology.

If something is well formed and already out there, it’s probably not for us. But we’ll fund anything—from high-risk projects that haven’t ever been tried before to those where quick iterative work is needed to get some cutting-edge technology into the hands of users even if there’s already some proof of concept.

## How will you prioritize which projects to fund?

Topically speaking, we want to be as diverse as possible in the type of capabilities we fund, whether it’s a software approach or a hardware one. But we are not prescriptive in what those need to be.

That’s really the whole point of the organization: to be a catalyst for many, many different components of health research that need help. And then the programs can go out to the other organizations that may have longer-term funding, but we brought down the risk for them.

[In 2022], the U.S. President asked for $6.5 billion to be made available to us for 3 years, so we are poised to definitely grow very quickly. But there’s no requirement that ARPA-H projects need to be centered in the U.S., and we know what a big challenge global health is. We’re all human. We tell ourselves: “The H is for humans.”

*Dalmeet Singh Chawla is a freelance contributor to*Chemical & Engineering News*, the independent news outlet of the American Chemical Society. Center Stage interviews are edited for length and clarity*

